# Vitamin D Receptor Gene Polymorphisms and the Risk of CIN2+ in Shanxi Population

**DOI:** 10.1155/2022/6875996

**Published:** 2022-11-16

**Authors:** Dongyan Li, Yan Liu, Dangyi Kong, Dimitri Papukashvili, Nino Rcheulishvili, Hongwei Zhao, Yinge Li, Chaiyun Hou, Jinfeng Ma, Xiaoqing Lu, Wenqi Bai

**Affiliations:** ^1^Shanxi Province Cancer Hospital/Shanxi Hospital Affiliated to Cancer Hospital, Chinese Academy of Medical Sciences/Cancer Hospital Affiliated to Shanxi Medical University, Taiyuan, China; ^2^The Second Hospital of Shanxi Medical University, Taiyuan 030001, China; ^3^Department of Pharmacology, School of Medicine, Southern University of Science and Technology, Shenzhen 518000, China; ^4^Department of Obstetrics and Gynecology, Shuozhou Central Hospital, Shanxi Province, China; ^5^Department of Obstetrics and Gynecology, Hequ County Hospital, Shanxi Province, China

## Abstract

Cervical cancer is one of the most common malignancies in women with high morbidity and mortality. Human papillomavirus (HPV) infection is the primary cause of cervical cancer, of which HPV 16 is the predominant. Early detection and effective treatment of cervical precancerous lesions are the key to preventing cervical cancer. Vitamin D receptor (VDR) gene polymorphism is considered to be an important cause of cancer development. Here, we studied the association of VDR polymorphisms (FOKI, BsmI, ApaI, and TaqI) in HPV16-positive cervical intraepithelial neoplasia (CIN)2+ patients. HPV16-positive patients who visited the Colposcopy Clinic of Obstetrics and Gynecology, the Second Hospital of Shanxi Medical University for biopsy due to abnormal HPV and/or Thinprep cytologic test (TCT) from September 1, 2020 to October 1, 2021 were grouped by pathological results. The fasting blood samples were collected and VDR polymorphisms were detected using TaqMan fluorescent probes, and the three sites of BsmI-ApaI-TaqI were subjected to haplotype analysis. FOKI ff genotype (OR = 2.01; 95% CI = 1.12 − 3.59; *p* = 0.019) and f allele (OR = 1.48; 95% CI = 1.10 − 1.98; *p* = 0.009) were found to be associated with the risk of CIN2+. TaqI Tt genotype (OR = 2.03; 95% CI = 1.20 − 3.43; *p* = 0.008), tt genotype (OR = 2.09; 95% CI = 1.09 − 4.02; *p* = 0.028), and t allele (OR = 1.35; 95% CI = 1.01 − 1.80; *p* = 0.041) were associated with the risk of CIN2+. No haplotype was associated with CIN2+ risk. According to the results, FOKI and TaqI polymorphisms are associated with CIN2+ risk.

## 1. Introduction

Cervical cancer is one of the common malignant tumors in gynecology, with high morbidity and mortality [1]. According to statistics, 604,000 new cervical cancer cases and 342,000 deaths were reported worldwide in 2020 [2], and about 85% of cervical cancers occur in developing countries. As the largest developing country in the world, the incidence of cervical cancer in China cannot be underestimated [3]. High-risk human papillomaviruses (HR-HPV) infection is the leading cause of cervical cancer, 70% of which are caused by HPV16/18, but only about 1% of persistent HR-HPV infection causes the occurrence of cervical cancer [4]. In addition, studies have shown that factors such as smoking, prolificity, and long-term oral contraceptives are associated with the occurrence of cervical cancer. Some scholars believe that the lack of nutrients such as vitamin D (VD) and folic acid in the diet can lead to the occurrence of cervical cancer [5].

Current studies have shown that VD deficiency is associated with many diseases, such as disorders of the immune, cardiovascular, respiratory, reproductive, and endocrine systems as well as malignancies such as prostate, colorectal, and breast cancers [6, 7]. Increasing evidence shows that VD can regulate the whole process from cancer development to metastasis and the related role of cells with the microenvironment. The specific mechanisms include regulating cell proliferation, differentiation, apoptosis, and autophagy as well as the regulation of angiogenesis, antioxidants, inflammation, and the immune system [8, 9]. VD is a lipid-soluble vitamin derived by sunlight or diet and is first delivered to the liver where it is metabolized into 25(OH)D and circulates in the serum. In the kidney it is further metabolized to the biologically most active form of VD—calcitriol (1*α*,25(OH)2D). It plays various roles in the body by binding to vitamin D receptors (VDR) [8]. VDR belongs to the nuclear receptor family with a typical nuclear receptor domain, including DNA-binding domain, a hinge region, a ligand-binding domain, and a carboxy-terminal activation function 2 domain that interacts with coregulators [10]. The VDR gene is located on chromosome 12q12-14, which contains two promoters and 8 exons [11]. So far, more than 60 VDR single nucleotide polymorphisms (single-nucleotide polymorphisms, SNPs) have been identified, but the most studied are FOKI (exon 2), BsmI, ApaI (intron 8), and TaqI (exon 9). The four SNP are located in different regions of the VDR gene and are potentially linked to many diseases [11, 12]. Many studies have analyzed the association of VDR SNP with cancer, such as breast cancer (FokI, BsmI, and ApaI), prostate cancer (FokI, BsmI, and TaqI), colorectal cancer (FokI, BsmI, and TaqI), and skin cancer (FokI, BsmI, and TaqI) [13], but there are still controversies. However, there are few studies on VDR SNPs, cervical precancerous lesions and carcinoma in China. The animal experimental results of Shim [14] showed that the treatment regimen of in situ immunization (CPG+OX40) combined with antiangiogenesis (anlotinib) increased the infiltration of CD4+ and CD8+ T cells in mice with cervical cancer, inhibited the growth of tumor volume, and prolonged the survival time of mice. These results indicate that immune dysfunction is an important mechanism leading to the development of cervical cancer. Chu found in his study that VD level was positively correlated with CD4 and negatively correlated with CD8 [15].

The occurrence of cervical cancer follows a gradual process from normal cervical to cervical intraepithelial neoplasia grade 1 (CIN)1 to CIN2/3 and finally develops into cervical cancer [16]. Only 1% of CIN1 cases progress to cervical cancer per year, while the risk of CIN2/3 progression to cervical cancer is 16% within 2 years and 25% within 5 years [17]. Therefore, the timely diagnosis and treatment of CIN2/3 is crucial to prevent the occurrence of cervical cancer. This project aimed to investigate the relationship between VDR SNPs (FOKI, BsmI, ApaI, and TaqI) and the risk of CIN2/3 and cervical cancer in Shanxi women. Additionally, serum concentration of 25-(OH)-D3 levels were detected in the VDR genotype difference group.

## 2. Materials and Methods

### 2.1. Subjects

The research samples were collected according to the HPV and/or Thinprep cytologic test (TCT) abnormalities from the subjects who visited the hospital from September 1, 2020 to October 1, 2021. The patients signed the informed consent, completed the questionnaire, and took fasting blood samples. Patients undergoing biopsy in the Colposcopy Clinic of the Obstetrics and Gynecology Department, the Second Hospital of Shanxi Medical University, Shanxi Provincial Cancer Hospital, Shuozhou Central Hospital, and Hequ County Hospital, Shanxi Province were selected as HPV16 positive patients, and the pathological results were used as the basis for grouping. The pathological results showed chronic inflammation as the control group and the CIN2+ (CIN2 and CIN3 and cervical cancer) as the case group. After screening according to the inclusion and exclusion criteria, 188 patients from the case group and the control group were separately grouped, among which 20 cases were included in Shanxi Cancer Hospital, and 6 cases were separately included in Shuozhou Central Hospital of Shanxi Province and Hequ County Hospital of Shanxi Province. The remaining cases were obtained from the Second Hospital of Shanxi Medical University.

### 2.2. Inclusion Criteria

All study subjects were of Han nationality, had lived in Shanxi for more than 5 years, age range from 22 to 65, were married or unmarried, and had at least two years of sexual life history. The exclusion criteria are as follows: patients with multiple systemic diseases such as immune, digestive, blood, and other malignancies; medication affecting vitamin D metabolism within 3 months before the study; pregnant women; patients with cervical adenocarcinoma. All study subjects signed informed consent and drew peripheral blood for analysis of VDR polymorphisms. Serum concentration of 25-(OH)-D3 levels were detected in the VDR genotype difference group. This study was performed with approval from the Ethics Committee of the Second Clinical Medical College of Shanxi Medical University.

### 2.3. Peripheral Blood DNA Extraction

Genomic DNA was extracted from peripheral blood according to the instructions of the QIAamp DNA Blood Mini (250) kit (Qiagen, Valencia, CA, USA). The concentration and purity of the extracted DNA samples were determined via NanoDrop one (Thermo Fisher Scientific, Waltham, MA, USA), and all the DNA samples that meet the standard were repackaged and stored at -80°C.

### 2.4. Detection of Serum 25-(OH)-D3

For the detection of serum 25-(OH)-D3, ELISA assay was used. The detection principle is as follows: the reagent used double antibody sandwich enzyme-linked immunosorbent assay. The procedure is as follows: (1) standard dilution, set the sample concentration gradient as follows: 25-(OH)-D3:200 ng/mL, 100 ng/mL, 50 ng/mL, 25 ng/mL, 12.5 ng/mL, 6.25 ng/mL, 3.13 ng/mL, 0 ng/mL; multiple wells were set for each concentration. (2) The same amount of standard/sample was added to be tested and biotinylated detection Ab (configured with the kit) and mixed well. (3) Plate was sealed and incubated at 37°C for 45 minutes. (4) It was mixed with washing liquid. (5) The plate was washed and pat dried 3 times. (6) HRP conjugate to each well was added and incubated at 37°C for 30 minutes. (7) The plate was washed and pat dried 5 times. (8) Substrate reagent was added to each well and incubated at 37°C for 15 min in dark. (9) The stop solution was added to stop the reaction. (10) The parameters of BioTekEPOCH2 microplate reader were set at 450 nm and. (11) The standard curve equation was calculated to obtain the sample concentrations.

### 2.5. Detection of the Vitamin D-Receptor Genetic Polymorphisms

All of the genomic DNA samples were typed for the following four SNP loci: FOKI (rs2228570), BsmI (rs1544410), ApaI (rs7975232), and TaqI (rs731236). SNP genotyping was performed using the TaqMan fluorescence probe method and ABI real-time PCR (Thermo Fisher Scientific, Waltham, MA, USA) instrument. Forty cycles were performed with the following operating conditions: denaturation step at 95°C for 10 min, denaturation step at 95°C for 15 sec, and annealing extension step at 60°C for 60 sec.

### 2.6. Statistical Analyses

All of the statistical analyses were performed using the SPSS 26.0 software (IBM). Counting data were expressed as the frequency and percentage, and comparisons between groups were performed using the chi-square (*χ*2) test. If the measurement data conform to the normal distribution, it is expressed by *t*, and the comparison between groups is expressed by the *t*-test. If not, it is expressed by M50 (25, 75) and the Mann–Whitney *U*-test is used for intergroup comparison. If the control genotype distribution complied with the Hardy-Weinberg equilibrium law *χ*2 test was used. The SHEsis software was used to perform linkage disequilibrium (LD) and haplotype analysis. The logistic regression analysis was applied to identify the association of the VDR SNPs or haplotypes and the risk of cervical lesions and to calculate the OR value and the 95% confidence interval (CI). *p* < 0.05 was defined as statistically significant.

## 3. Results

The basic data of all the study subjects are presented in Table 1. The age of the case and control groups was, respectively, 47.77 ± 7.65 and 45.07 ± 7.93 years, which was statistically significant (*p* < 0.05). In addition, the age at first sex, menopause, and smoking were statistically significant in the case group when compared with the controls (*p* < 0.05).

**Figure 1:**
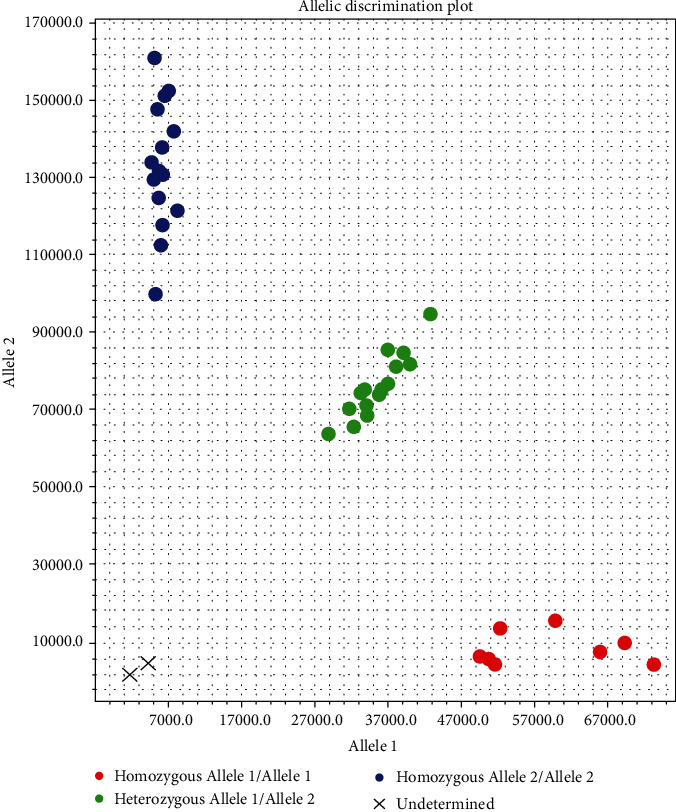
TaqMan genotyping results; X represents blank control.

The results of the partial TaqMan genotyping analysis are given in Figure 1. The distribution of the VDR SNP genotypes and alleles in both groups and the relationship with the risk of developing CIN2+ are listed in Table 2.

For the FOKI polymorphisms, the allele f was more common in the CIN2+ group (64.63%) than in the control group (55.32%). Comparing the Ff genotype with the FF genotype showed that the Ff genotype was not associated with CIN2+ risk (crude OR = 1.38; 95% CI = 0.78 − 2.43; *p* = 0.273 and adjusted OR = 1.47; 95% CI = 0.81 − 2.67; *p* = 0.205). Comparing the ff genotype with the FF genotype showed that the ff genotype significantly increased the risk of CIN2+ occurrence (crude OR = 2.01; 95% CI = 1.12 − 3.59; *p* = 0.019 and adjusted OR = 2.18; 95% CI = 1.19 − 3.99; *p* = 0.012). After adjusting for the confounders, f allele carriers were not associated with CIN2+ risk (adjusted OR = 1.77; 95% CI = 1.02 − 3.08; *p* = 0.041). Finally, by comparing the f allele to the F allele, the f allele was associated with an increased CIN2+ risk (OR = 1.48; 95% CI = 1.10 − 1.98; *p* = 0.009).

For the TaqI polymorphisms, the allele t was more common in the CIN2+ group (52.39%) than in the control group (44.95%). The Tt, tt genotypes were separately compared to the TT genotype, the results showed that the Tt and tt genotypes significantly increased the risk of CIN2+ (Tt VS TT: crude OR = 2.03; 95% CI = 1.20 − 3.43; *p* = 0.008 and adjusted OR = 1.94; 95% CI = 1.12 − 3.35; *p* = 0.018. tt VS TT: crude OR = 2.09; 95% CI = 1.09 − 4.02; *p* = 0.028 and adjusted OR = 1.98; 95% CI = 1.17 − 3.37; *p* = 0.012). Comparison of the Tt + tt genotype with the TT genotype showed that the t allele carriers are associated with CIN2+ risk (crude OR = 2.04; 95% CI = 1.23 − 3.40; *p* = 0.006 and adjusted OR = 1.98; 95% CI = 1.17 − 3.37; *p* = 0.012). Finally, comparing the t allele to the T allele revealed that the t allele is a CIN2+ susceptibility gene (OR = 1.35; 95% CI = 1.01 − 1.80; *p* = 0.041). The BsmI and ApaI polymorphisms were not associated with CIN2+ risk.

**Figure 2:**
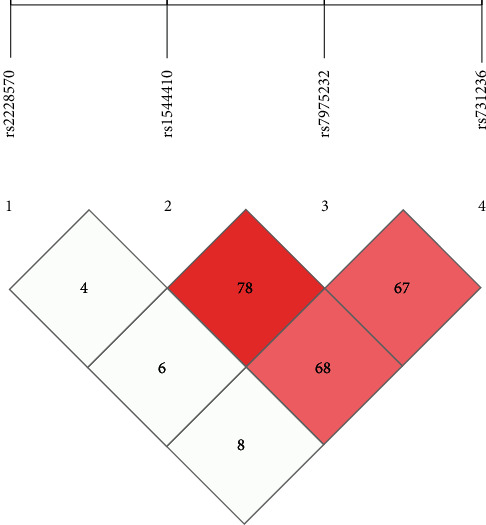
Linkage disequilibrium map of the four SNP species for all the study subjects. The percentage of D' is indicated by the strength of numbers and color.

The SHEsis software was applied to create an LD diagram (Figure 2). As the LD between the FOKI polymorphism and the other SNP was very low, it was excluded, and the haplotypes of the other three SNPs were analyzed. The results of this study showed that the baT haplotypes were the most common in both patients and controls, and that the other haplotypes were not significantly different when compared to the baT. To further observe the genotype distribution across all study subjects, the frequency of the combined BsmI, ApaI, and TaqI genotypes in the CIN2+ and control groups were analyzed (Table 3). As the distribution of BbAaTt was the most common in controls, it was selected as the reference genotype. However, further analysis in this study showed that none of the combined genotypes was associated with the risk of CIN2 +.

Serum concentrations of 25-(OH)-D3 were detected in the VDR FOKI polymorphisms.

The results are different, 25-(OH)-D3 concentration was different among different genotypes in the control group, and the relative 25-(OH)-D3 concentration of F gene was higher among different groups (*p* < 0.05). The relative 25-(OH)-D3 concentration decreased in both F and F genome in HSIL group and SCC group, which was significantly different from that in the control group (*p* < 0.05). There was no significant difference among the groups (Table 4).

The main effect analysis showed that there were interaction effects between groups and genotypes (*F*interaction = 3.040, *p* = 0.017), so further separate effect analysis was needed (Table 5).

## 4. Discussion

Due to the increase of new cases and deaths caused by cervical cancer, this cancer may become one of the greatest burdens of oncology, thus, further identification of the factors affecting the development of cervical cancer is crucial. This study analyzed the relationship between VDR polymorphism and HPV16-positive CIN2+ patients in Shanxi women, aiming to provide a theoretical basis for reducing the occurrence of cervical cancer.

VDR is a member of the steroid/thyroid hormone receptor family that regulates biological processes including cell proliferation, differentiation, apoptosis, tumor invasion, and angiogenesis in vivo [18, 19]. VDR polymorphisms themselves may not be genetic loci affecting disease progression, but rather play a role by affecting VDR expression levels [20]. Studies of the VDR polymorphisms suggest that the VDR polymorphism may be an important factor affecting the VDR mRNA and protein numbers and affecting the downstream VD-mediated effects [20]. More than 60 VDR SNPs, located in the promoter regions of exons 2–9, have been linked to the occurrence and prognosis of cancer [12]. However, only a few of them, including FOKI, BsmI, ApaI, and TaqI, have potential functions to affect the expression of VDR genes and are associated with cancer risk [12].

Regarding the FOKI polymorphism, since the ff genotype and the f allele increase the incidence of CIN2+, the present results indicate that the f allele is a susceptibility allele affecting the occurrence of cervical lesions, in line with previous studies in the Thai population [21]. In addition, the f allele is also a susceptibility gene for T cell lymphoma [22], colorectal cancer [12], and ovarian cancer [23]. The FOKI f allele is associated with an increased risk of cervical cancer and most other cancers, possibly due to its reduced VDR activity [22]. However, the meta-analysis of Pu et al. [9] showed that the incidence of head and neck cancer in patients with ff genotype was significantly lower than that in patients with Ff+FF or FF genotype. The FOKI SNP is located in the second exon, and involves the translation initiation point, forming an additional start codon by changing the ACG codon located ten base pairs upstream of the translation start codon [22, 24]. Therefore, two variants of the VDR protein may occur during translation: a longer form of the protein (f allele, containing 427 amino acids) and a shorter form (F allele, containing 424 amino acids) [22, 25]. Studies have shown that the longer form of the VDR protein has lower transcriptional activity, reducing the anticancer properties of calcitriol, and leading to increased cancer susceptibility [21, 22]. Therefore, this study supports the role of the FOKI f allele in increasing susceptibility to most cancers.

Regarding the TaqI polymorphism, the results of this study showed that the t allele affects the risk of the CIN2+ occurrence, consistent with the findings of Phuthong et al. [21]. However, the present study demonstrated that the ApaI and BsmI polymorphisms were not associated with CIN2+ risk. Previous studies have shown that the BsmI polymorphism increases the risk of gastric cancer [19], colorectal cancer [12], melanoma [26], and multiple myeloma [18]. Moreover, Qadir et al. [19] proposed that the BsmI polymorphism follows the “law of dominant inheritance”, in which people with the BB genotypes have a lower risk of cancer than those with both the Bb and bb genotypes. In addition, the A allele carriers of the ApaI polymorphism increase the risk of colorectal cancer [12] and multiple myeloma [18], and instead reduce the risk of hepatocellular carcinoma caused by HCV infection [27]. Conversely, the t allele of the TaqI polymorphism is a susceptibility gene for colorectal cancer [12], and it is also an important factor in reducing the incidence of head and neck cancer as well as tobacco-related respiratory and oesophageal cancer [28].

The BsmI, ApaI, and TaqI polymorphisms are located in the 3′-noncoding region of the VDR genes (3′-untranslated, 3′UTR), and the three SNP species are in strong linkage disequilibrium with each other [19]. These three SNPs do not change the amino acid sequence of proteins but can regulate gene expression, especially mRNA stability [22, 29]. BsmI and ApaI polymorphisms are located in intron 8 of the VDR gene, resulting in silent mutations [30]. One of the mechanisms by which these two SNPs affect VDR expression is that the splicing site of VDR mRNA transcription is disrupted, resulting in truncation or alternate splicing of the protein product; another explanation is the altered polyadenylation of VDR mRNA and thus altered mRNA stability [19]. TaqI SNP, located in exon 9 of the VDR gene, is a synonymous SNP that results in a silent mutation in codon 352 from ATT to ATC, both of which encode isoleucine [29, 30]. In addition, the 3′ TaqI SNP has been found to affect CpG (where the TaqI SNP is located) methylation and CGI 1060 methylation [19]. CGI 1060 is located at the 3′ end of the VDR, and its methylation may affect the regulation of the 3′ promoter, driving lncRNA transcription in this region, and may regulate the expression of the VDR posttranscriptionally [19].

Studies of haplotypes and combined genotypes can provide more conclusive information about genetic variation, however, in this study, no single haplotype or combined genotype was found to be associated with CIN2+ risk. In a meta-analysis by Khan et al. [24] it was shown that bAT haplotypes are more common in African populations (59%), whereas baT and BAt haplotypes are more common in Asian (75%) and Caucasian populations (39%). In the current study, the baT haplotype was the most frequent in both patients and controls, which is consistent with the results of Khan et al. [24]. In another study, the baT haplotype was found to be the most common in colorectal cancer [31]. Gleba et al. [22] suggested that the baT haplotype increases the sensitivity of leukemia and lymphoma cells to calcitriol, and Gleba believes that the baT haplotype is associated with increased VDR mRNA transcriptional activity, which can lead to the formation of more many VDR proteins. Latacz et al. [31] found that tAb and Bat haplotypes increased the risk of colorectal cancer (tAb: OR = 3.84; 95% CI 1.29-11.38; *p* = 0.01, Bat: OR = 30.22; 95% CI 2.81 -325.31; *p* = 0.01). However, Qadir et al. [19] suggested that the ATC (bat) haplotype increases the risk of gastric cancer, while the GTT (BaT) haplotype plays a protective role. Different haplotypes are expressed differently in different cancers, possibly due to differences in gene-environment interactions and lifestyles. Large differences in VDR genotypes by ethnicity have been demonstrated [32]. The results of this study demonstrated the following: 25-(OH)-D3 in the control group indicates that the F-gene population requires relatively high 25-(OH)-D3 concentrations under normal conditions. 25-(OH)-D3 concentration decreases and the disease progresses. Combined with FOKI polymorphisms analysis, F gene is a risk factor for CIN2+ disease, and the decrease of 25-(OH)-D3 concentration in F gene population suggests an increased risk of CIN2+ disease. In addition, geographic differences and a limited number of studies may also contribute to this disparity. To our knowledge, this study is the first to report the relationship between VDT polymorphisms and the risk of HPV 16-positive CIN2+ disease in the Shanxi Province population. In this study, only HPV 16-positive population was included while the other high-risk HPV-infected populations were not included, which cannot represent all high-risk HPV-infected populations. This is the limitation of the study and the addition of other high-risk HPV-infected populations to verify the results in the subsequent studies are warranted.

## 5. Conclusions

This study is the first to demonstrate the role of the VDR polymorphism in HPV16-positive cervical lesions in Shanxi women, and the FOKI and TaqI genotypes may be associated with a high risk of CIN2+. However, no single haplotype was found to increase the CIN2+ risk in our study. The binding ability of the VDR to its target sequence may be influenced by specific DNA sequences, VDR isoforms, cell-specific phosphorylation, and changes in core regulators in different tissues [9]. Therefore, the underlying mechanisms of different VDR gene polymorphisms in cervical cancer remain to be further investigated.

## Figures and Tables

**Table 1:** Basic data for all of the study subjects.

Variable	Cases (*n* = 188)	Controls (*n* = 188)	*t*/*z*/*χ*2Value	*p* value
Age	47.77 ± 7.65	45.07 ± 7.93	3.357	<0.001∗
Degree of education				
Illiteracy	10 (5.32)	7 (3.72)	6.021	0.304
Primary school	30 (15.96)	21 (11.17)		
Junior middle school	66 (35.11)	60 (31.91)		
Senior middle school	28 (14.89)	35 (18.62)		
Junior college	30 (15.96)	28 (14.89)		
Bachelor's degree or above	24 (12.77)	37 (19.68)		
Occupation				
Medical personnel	7 (3.72)	7 (3.72)	12.414	0.134
Science and education workers	14 (7.45)	7 (3.72)		
Administration staff	5 (2.66)	16 (8.51)		
Worker	15 (7.98)	7 (3.72)		
Farmer	24 (12.77)	27 (14.36)		
Commerce	14 (7.45)	10 (5.32)		
Service	14 (7.45)	15 (7.98)		
Others	56 (29.79)	63 (33.51)		
Unemployed	39 (20.74)	36 (19.15)		
Smoking				
Yes	49 (26.06)	26 (13.83)	8.811	0.003^∗^
No	139 (73.94)	162 (86.17)		
Husband smoking				
Yes	130 (69.15)	126 (67.02)	0.196	0.658
No	58 (30.85)	62 (32.98)		
Age of menarche	14.38 ± 1.82	14.41 ± 1.69	-0.176	0.860
Menopause				
Yes	111 (59.04)	72 (38.30)	16.192	<0.001∗
No	77 (40.96)	116 (61.70)		
Marriageable age	22.76 ± 2.91	22.83 ± 3.09	-0.240	0.810
Age at first sex life	21.85 ± 2.84	22.49 ± 3.07	-2.113	0.035∗

(∗*p* < 0.05, the difference was statistically significant).

**Table 2:** Distribution of VDR SNP genotypes versus alleles in the case and control groups.

Genotype/allele	Case *n* (%)	Control *n* (%)	Crude OR (95% CI)	*p* value	Adjusted OR^b^ (95% CI)	*p* value
FOKI^a^						
FF	28 (14.89)	42 (22.34)	—	—	—	—
Ff	77 (41.96)	84 (44.68)	1.38 (0.78-2.43)	0.273	1.47 (0.81-2.67)	0.205
ff	83 (44.15)	62 (32.98)	2.01 (1.12-3.59)	0.019	2.18 (1.19-3.99)	0.012
Ff+ff	160 (85.11)	146 (77.66)	1.64 (0.97-2.79)	0.065	1.77 (1.02-3.08)	0.041
F	133 (35.37)	168 (44.68)	—	—	—	—
f	243 (64.63)	208 (55.32)	1.48 (1.10-1.98)	0.009	—	—
BsmI^a^						
BB	15 (7.98)	18 (9.57)	—	—	—	—
Bb	61 (32.45)	69 (36.70)	1.06 (0.49-2.28)	0.880	0.92 (0.41-2.08)	0.845
bb	112 (59.57)	101 (53.73)	1.33 (0.64-2.78)	0.447	1.25 (0.58-2.71)	0.571
Bb+bb	173 (92.02)	170 (90.43)	1.22 (0.60-2.50)	0.585	1.12 (0.53-2.39)	0.762
B	91 (24.20)	105 (27.93)	—	—	—	—
b	285 (75.80)	271 (72.07)	1.21 (0.88-1.68)	0.245	—	—
ApaI^a^						
AA	22 (11.70)	31 (16.49)	—	—	—	—
Aa	92 (48.94)	85 (45.21)	1.53 (0.82-2.84)	0.183	1.38 (0.72-2.66)	0.227
aa	74 (39.36)	72 (38.30)	1.45 (0.77-2.74)	0.253	1.34 (0.68-2.63)	0.394
Aa+aa	166 (88.30)	157 (83.51)	1.49 (0.83-2.68)	0.184	1.36 (0.73-2.54)	0.332
A	136 (36.17)	148 (39.36)	—	—	—	—
a	240 (63.83)	228 (60.64)	1.15 (0.85-1.54)	0.367	—	—
TaqI^a^						
TT	29 (15.43)	51 (27.13)	—	—	—	—
Tt	121 (64.36)	105 (55.85)	2.03 (1.20-3.43)	0.008	1.94 (1.12-3.35)	0.018
tt	38 (20.21)	32 (17.02)	2.09 (1.09-4.02)	0.028	2.14 (1.07-4.28)	0.031
Tt+tt	159 (84.57)	137 (72.87)	2.04 (1.23-3.40)	0.006	1.98 (1.17-3.37)	0.012
T	179 (47.61)	207 (55.05)	—	—	—	—
t	197 (52.39)	169 (44.95)	1.35 (1.01-1.80)	0.041	—	—

^a^ All genotypes in the control group followed the Hardy-Weinberg equilibrium law. ^b^ Multivariate logistic regression adjusting for age, smoking, menopause, and age at first sex).

**Table 3:** The distribution of three SNP haplotypes: BsmI, ApaI, and TaqI.

Haplotype	Case frequency (%)	Control frequency (%)	*p* value
baT	45.5	42.4	—
BAT	1.0	4.1	0.339
Bat	21.1	19.1	0.939
BaT	0	2.5	0.331
Bat	2.1	2.2	0.906
bAT	1.1	6.0	0.142
bAt	13.0	10.2	0.714
bat	16.2	13.5	0.782
BbAaTt	44 (23.40)	46 (24.47)	—
bbaaTt	38 (20.21)	31 (16.49)	0.440
bbAaTt	35 (18.62)	22 (11.70)	0.139
BBAAtt	12 (6.38)	9 (4.79)	0.497
bbaaTT	28 (14.89)	31 (16.49)	0.864
BbAATT	1 (0.53)	3 (1.60)	0.369
BbAatt	10 (5.32)	8 (4.26)	0.606
BBAATT	0	2 (1.06)	—
BBAATt	1 (0.53)	1 (0.53)	—
Bbaatt	3 (1.60)	3 (1.60)	—
BBAaTT	0	2 (1.06)	—
bbAatt	3 (1.60)	3 (1.60)	—
BBaatt	2 (1.06)	2 (1.06)	—
BBaaTT	0	2 (1.06)	—
BbAATt	3 (1.60)	3 (1.60)	—
BbAAtt	0	2 (1.06)	—
BbAaTT	0	2 (1.06)	—
bbAAtt	5 (2.66)	5 (2.66)	—
BbaaTT	0	2 (1.06)	—
bbAaTT	0	3 (1.60)	—
bbAATT	0	4 (2.13)	—
bbAATt	0	2 (1.06)	—
bbaatt	3 (1.60)	0	—

**Table 4:** Main effect analysis of serum concentration of 25-(OH)-D3.

Effect class	III square sum mean square of	Degrees of freedom	The mean square	*F*	*p*
Correction model	31330.029	8	3916.254	28.558	<0.001
Intercept	171976.908	1	171976.908	1254.101	<0.001
Group	21483.815	2	10741.907	78.333	<0.001
Genotype	61.221	2	30.61	0.223	0.800
Group ^∗^ genotype (interaction)	1667.494	4	416.874	3.040	0.017
Error	50327.3	367	137.132		

**Table 5:** Separate effect analysis of serum concentration of 25-(OH)-D3.

Genotype	Control	HSIL group	SCC group	*F*	*p*
FF	39.93 ± 14.92	27.74 ± 6.92&	24.00 ± 9.61&	9.003	<0.001
Ff	46.96 ± 12.86∗	26.00 ± 8.98&	20.66 ± 8.22&%	81.000	<0.001
ff	40.13 ± 14.05#	27.09 ± 10.09&	23.13 ± 9.85&	24.855	<0.001
*F*	5.883	0.377	0.432		
*P*	0.003	0.686	0.652		

^∗^ indicates statistical difference compared with FF, # indicates statistical difference compared with FF, & indicates statistical significance compared with the control group, and % indicates statistical significance compared with the HSIL group.

## Data Availability

The data presented in this study are available on request from the corresponding author.
